# Inequalities in the Use of Family Planning in Rural Nepal

**DOI:** 10.1155/2014/636439

**Published:** 2014-08-28

**Authors:** Suresh Mehata, Yuba Raj Paudel, Bhogendra Raj Dotel, Dipendra Raman Singh, Pradeep Poudel, Sarah Barnett

**Affiliations:** ^1^Nepal Health Sector Support Programme, Ministry of Health and Population, Ramshah Path, Kathmandu 44600, Nepal; ^2^Karuna Foundation Nepal, Baluwatar, Kathmandu 44616, Nepal; ^3^Ministry of Health and Population, Ramshah Path, Kathmandu 44600, Nepal; ^4^Options Consultancy Services Limited, Devon House, 58 St Katharine's Way, London E1W 1LB, UK

## Abstract

This paper explores inequalities in the use of modern family planning methods among married women of reproductive age (MWRA) in rural Nepal. Data from the 2012 Nepal Household Survey (HHS) were utilized, which employed a stratified, three-stage cluster design to obtain a representative sample of 9,016 households from rural Nepal. Within the sampled households, one woman of reproductive age was randomly selected to answer the survey questions related to reproductive health. Only four out of every ten rural MWRA were using a modern family planning method. Short-acting and permanent methods were most commonly used, and long-acting reversible contraceptives were the least likely to be used. Muslims were less likely to use family planning compared to other caste/ethnic groups. Usage was also lower among younger women (likely to be trying to delay or space births) than older women (likely to be trying to limit their family size). Less educated women were more likely to use permanent methods and less likely to use short-term methods. To increase the CPR, which has currently stalled, and continue to reduce the TFR, Nepal needs more focused efforts to increase family planning uptake in rural areas. The significant inequalities suggest that at-risk groups need additional targeting by demand and supply side interventions.

## 1. Introduction

In Nepal, the National Health Policy (1991), the Second Long-Term Health Plan (1997–2017), and the National Reproductive Health Strategy (1998) have all emphasized the need to improve equitable access to quality reproductive health services. Since 2010, it has been government policy to provide at least five different family planning methods at all levels of health facility from health post and above [1]; however, just 8% of health posts have met this target [2]. In the community, injectable contraceptives are available from Community Health Workers (CHWs), and Female Community Health Volunteers (FCHVs) undertake educational and promotional activities on family planning and distribute oral contraceptive pills and condoms [1].

Many barriers prevent the use of family planning and result in unplanned pregnancies [3]. These barriers are multifactorial, including both client-related factors such as a lack of education and exposure to media resulting in poor knowledge about family planning methods and services [3], low economic status [3], and concerns and experience of side effects [3] and health system factors such as poor coverage of health facilities [4], lack of outreach services [4], stock-outs and poor method mix [3], limited providers and poor provider competence [4], and lack of advice and counselling [4]. Both client- and system-related barriers contribute to increased inequality in the utilization of family planning services.

**Figure 1:**
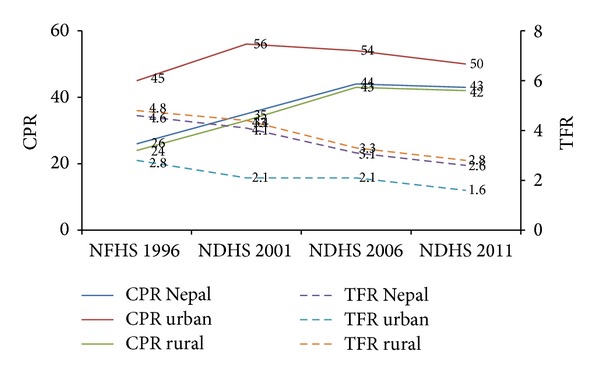
CPR and TFR in Nepal, 1996–2011, disaggregated by urban and rural residence. Sources:* Nepal Family Health Survey 1996; Nepal Demographic Health Survey 2001; Nepal Demographic Health Survey 2006; and Nepal Demographic Health Survey 2011*.

Between 1996 and 2006 the national contraceptive prevalence rate (CPR) increased by 69% in Nepal, from 26% in 1996 [5] to 44% in 2006 [6], but between 2006 and 2011 it stalled [7] (Figure 1). Similarly, the CPR in rural areas increased from 24% in 1996 [5] to 42% in 2006 [6] but then stalled between 2006 and 2011. A different pattern was observed in urban areas: the CPR increased from 45% in 1996 to 56% in 2001 [8] but then declined to 50% in 2011 [7]. Despite the stalled CPR in rural areas and the decreased CPR in urban areas, the TFR has continued to decline in both: from 2.8 in 1996 [6] to 1.6 in 2011 [7] in urban areas and from 4.8 in 1996 [6] to 2.8 in 2011 [7] in rural areas. However, the decline in the TFR may be due to the momentum gained in last decade, and if substantial efforts are not put in place now to increase the CPR it is unlikely that this decline will continue.

Given that most of the Nepalese population live in rural areas (83%), the national CPR and total fertility rate (TFR) are aligned to the rural figures and a better understanding of inequalities in family planning use within rural areas is required to inform efforts to meet the national MDG target for the TFR of 2.5. Many studies have documented lower availability and use of family planning methods in rural areas [9, 10] and inequalities between different groups [11]. Rural women often have lower levels of education and lower socioeconomic status, which may reduce access to family planning [12], and input into decision making [13].

The objective of this paper is to assess the prevalence and inequalities (by age, level of education, economic status, caste/ethnicity, access to health facility, and ecological zone) in the use of modern family planning methods, among married women of reproductive age in rural Nepal.

## 2. Methodology

### 2.1. Study Design and Sampling

This paper used data collected between August and September 2012 for the nationally representative, cross-sectional 2012 Nepal Household Survey (HHS 2012) coordinated by authors of this paper, in collaboration with the Ministry of Health and Population (MoHP). The primary objective of the survey was to provide national estimates for key reproductive, maternal, neonatal, and child health indicators [14]. Since some of the survey questions related to the uptake of family planning, the MoHP was keen to see further analysis of these data to explore inequalities in family planning in rural Nepal.

Nepal consists of 75 districts divided into three ecological zones (mountain, hill, and Terai), five administrative regions, and 13 subregions. Districts are divided into village development committees (VDCs) (considered to be rural) and municipalities (considered to be urban). These in turn are divided into wards, with each VDC having nine wards. A stratified, three-stage cluster design was employed in the HHS 2012, first selecting districts, then wards, and then households. Districts were the primary sampling units (PSUs), and one PSU was randomly selected from each of the 13 subregions. This resulted in three districts being selected from the mountain zone, five from the hill zone, and five from the Terai zone. Within these 13 PSUs, wards were used as the basis for clusters, and 180 clusters were selected with probability proportionate to size (PPS) (based on the number of households as per the National Population and Housing Census 2011) [15]. From each cluster, 57 households were selected using systematic sampling to obtain a representative sample of 10,260 households of which 9,016 were in rural areas. Within the sampled households, one woman of reproductive age (15–49 years) was randomly selected to answer the survey questions related to reproductive health. However, the current analysis is based on responses from 7442 married women of reproductive age (15–49 years) from the rural households. A more detailed description of the sampling methodology is presented in the HHS 2012 report [14].

### 2.2. Data Entry and Coding

All questionnaires were checked by a supervisory level at the time of data collection and coding of data was undertaken prior to data entry. All data were double entered into a CSPro 4.0 database and any inconsistencies were corrected. Data entry was closely supervised by a data manager. Prior to analysis data were checked for any anomalies and, where necessary, data were cross-checked with the original questionnaires.

### 2.3. Variables Included

The main outcome variable was the use of any modern contraceptive. This was broken down into permanent, long-acting, and short-acting methods, and these categories were used in the multinomial logistic regression analysis as outcome variables. The use of these broader categories, as opposed to showing results by individual method, is more relevant for government efforts to improve service delivery. Three levels of predictor variables were available from the HHS 2012 and were included in the analysis: individual (mother's age: 15–24 years, 25–34 years, and 35–49 years; maternal education: never attended school to higher education); household (caste/ethnic group categorized based on the classification recommended by Bennett et al. [16]; wealth quintile and distance to health facilities: less than 30 minutes, 30–60 minutes, and more than 60 minutes), and community (ecological zone: mountain, hill, and Terai).

### 2.4. Data Analysis

All analyses in this paper were conducted using STATA 12 SE Version. Prevalence values were weighted by sample weights to provide population estimates. The prevalence and 95% confidence intervals (95% CI) were calculated taking into consideration the complex survey design of the HHS 2012. The crude and adjusted odds ratios were assessed through binomial and multinomial logistic regression to estimate the inequalities, and a *P* < 0.05 was considered as statistically significant. All of the predictors (mother's age, education, economic status, caste/ethnicity, distance to health facilities, and ecological zone) were used in the final adjusted model.

## 3. Results

### 3.1. Any Modern Methods

Forty-one percent of MWRA in rural Nepal were using a modern family planning method (Table 1). The uptake of modern family planning methods increased with age (Table 2). Compared to Brahmins/Chhetris, Newars were nearly twice as likely (AOR: 1.9; 95% CI: 1.4–2.7) to use a modern method while Muslims and Terai Madhesi other castes were least likely. Women residing in hill districts were less likely to use a modern method than those in the mountain districts (AOR: 0.7; 95% CI: 0.5–0.9). No significant differences in the use of modern methods were noted between wealth quintiles, levels of education, and the time taken to reach the nearest government health facility. The use of short-term (21%) and permanent methods (18%) was far higher than the use of long-acting reversible contraceptives (LARCs) (2%) (Table 1).

### 3.2. Permanent Methods

Permanent methods (18%) were the second most commonly used group of modern methods. The likelihood of using permanent methods increased with age (as attainment of desired family size increases with age) and was more common among those who had never been to school. The multinomial regression supported this finding showing that the likelihood of using a permanent method decreased with increasing education level (Table 2). Permanent methods were most likely to be used in the Terai (23%) and the least likely to be used in the hill districts (12%). There were large differences in the use of permanent methods by caste/ethnic group, with Terai Madhesi other castes having the highest use (27%) and Muslims the lowest use (4%) (Table 1). The multinomial logistic regression analysis showed low use of permanent methods among Janajatis (AOR: 0.7; 95% CI: 0.5–0.9) and Muslims (AOR: 0.1; 95% CI: 0.1-0.2) compared to Brahmins/Chhetris. No significant differences were observed in wealth quintile, time taken to the nearest government health facility, or ecological zone (Table 2).

### 3.3. Long-Acting Reversible Methods

Use of LARCs (implants and IUCDs) was very low, with around 2% of MWRA using each method. Use of LARCs increased slightly for each age-group up to the peak use at 35–49 years (Table 1). The multinomial logistic regression analysis also showed use of LARCs to be higher among those aged 35 years or above compared to those aged below 25 (AOR: 2.0; 95% CI: 1.2–3.5). Use of LARCs among Terai/Madhesi other castes (AOR: 0.2; 95% CI: 0.1–0.7) was lower than among Brahmins/Chhetris (Table 2). Use of LARCs was slightly higher among those who lived less than 60 minutes travel time from a government health facility in comparison to those living more than 60 minutes away (Table 1). Multinomial logistic regression analysis also showed that use of LARCs was lower among those who resided more than 60 minutes away from their nearest government health facility (AOR: 0.5; 95% CI: 0.3–0.9) in comparison to those living less than 30 minutes away. No significant association was noted by ecological zone, wealth quintile, or level of education (Table 2).

### 3.4. Short-Term Methods

Short-term methods (21%) were the most commonly used group of methods of contraception. MWRA aged 25–34 years were most likely to use short-term methods (24%) among all age-groups, and those living in mountain (25%) or hill (25%) districts were more likely to use them compared to those living in Terai districts (15%). Use of short-acting methods was the lowest for those who had never attended school (17%) and the highest amongst the most educated (30%). This was supported by the multinomial logistic regression analysis, which showed that women with higher education were nearly twice as likely (AOR: 1.8; 95% CI: 1.1–2.6) to use a short-acting method compared to those who never attended school. The likelihood of using a short-acting method was higher among Newars and Janajatis than Brahmins/Chhetris, while Muslims were less likely to use short-acting method (Table 2). Those in the highest wealth quintile (23%) were more likely to use short-acting methods compared to other wealth quintiles (Table 1). No significant association was observed with time taken to reach the nearest government health facility (Table 2).

## 4. Discussion

In rural Nepal the challenging topography and lack of road infrastructure and transportation mean that many have to walk long distances over difficult terrain to reach health facilities. The 2012 HHS found that just over half of the rural population (53%) were within half an hour travel time of their closest health facility, compared to 80% in urban areas [14]. Distance to the nearest health facility has been identified as a barrier to family planning uptake in other studies [12, 17]. A study in Bangladesh revealed that couples who resided more than 30 minutes travel time from a facility were 25% less likely, and those living between 15 and 30 minutes were 20% less likely, to use FP methods in comparison to women who lived at a distance of less than 15 minutes [18]. However, except for LARCs, no significant association was found in this study between distance to a health facility and use of modern family planning methods. This may be partly attributed to the increased availability of family planning methods (injectables, pills, and condoms) at community level through outreach clinics, private pharmacies, and FCHVs, whereas use of LARCs, which are only available at facilities, decreased with increasing distance. Male sterilization was higher among those living further from a government health facility (data not shown). Males often use mobile camps for sterilization [7, 19, 20] and may be more likely to opt for sterilization if they face greater difficulties in accessing services for other family planning methods.

Use of female sterilization was most common among those living in the Terai, while male sterilization was least common in the Terai (data not shown). Male sterilization is sometimes believed to lead to impurity and exclusion from rituals and also cause physical weakness [21], but it is not clear whether this belief is more common in the Terai. Newars were most likely to use family planning methods and Muslims and Terai Madhesi other castes were least likely to use family planning methods. Similar findings have been reported in other studies [22]. Caste-based discrimination has been reported by Dalits, Muslims, and Terai Madhesi other castes at health facilities in regard to reduced access to care, delayed care, and poor quality of care, including reluctance by service providers to touch Dalits leading to fewer physical examinations and discourteous behaviour [23]. Continuing social exclusion also results in families not visiting health facilities to avoid potential discrimination and poor quality care [23–25].

Studies have reported increased use of family planning with increased education [25–27]. This paper showed that the use of permanent methods (male and female) was greater among those who have never attended school. This may reflect the higher use of sterilization among older couples, as they have already attained their desired family size, who were less likely to have attended school [7]. The use of short-term methods was almost double among those who had higher education compared to those who had never attended school. Use of contraceptives increased with age, contrary to the NDHS, which found that use was lower among younger and older women [7]. Other studies have reported higher contraceptive use among those with higher economic status [25–27], but this paper did not show a significant association.

The findings from current analysis present some implications for policy and future research. First, since use of LARCs has been found to be significantly associated with distance between health facility and home, women residing far away need to be reached through satellite, mobile, or outreach clinics [28] or by supplying LARCs through lower-level health facilities. Furthermore, short-term family planning methods can also be promoted through these clinics [20]. This could increase the CPR in rural areas because LARCs, especially implants, are becoming popular among MWRA in rural Nepal [29]. Second, efforts should also be made to increase FP use by Muslims, Dalits, and Terai Madhesi other castes. One approach to increase FP adoption could be to train the same caste health workers [30] to provide FP services in such areas or to orientate existing health workers in accountability and interpersonal communication. Third, given the link between education and use of family planning, female education and women's empowerment should be high on the agenda. Fourth, interaction with health providers during antenatal, delivery, postnatal care, and child health visits is an ideal opportunity to promote FP use, especially given that younger women had significantly higher unmet need for spacing during 2 years postpartum [31].

## 5. Conclusions

To increase the national CPR, which has currently stalled, and to ensure that the TFR continues to decline, additional efforts need to be focused on rural Nepal, including addressing the significant inequalities that exist. The findings from this paper suggest that efforts to supply LARCs within 30 minutes walking distance from homes in rural areas are likely to increase uptake. High risk groups, with lower use of family planning, such as Muslim, younger, and less educated women, need additional targeting by demand and supply side interventions.

## Figures and Tables

**Table 1:** Prevalence of use of any modern method, permanent method, long-acting and short-acting family planning methods by married women of reproductive age (MWRA) residing in rural areas.

	Not using any method		Any modern method		Permanent method		Long-acting reversible method		Short-acting method		Total no. of MWRA in rural areas
	*N*	% (95% CI)	*N*	% (95% CI)	*N*	% (95% CI)	*N*	% (95% CI)	*N*	% (95% CI)	
All MWRA	**4172**	**56.0 (53.2–58.9)**	**3033**	**40.7 (37.9–43.7)**	**1330**	**17.9 (15.6–20.4)**	**169**	**2.3 (1.7–3.1)**	**1534**	**20.6 (18.2–23.2)**	**7442**
Age-group											
15–24 years	1315	71.7 **(68.0–75.1)**	423	23.1 **(19.6–26.9)**	27	1.5 **(0.8–2.5)**	27	1.4 **(0.9–2.3)**	370	20.2 **(17.0–23.8)**	1833
25–34 years	1591	54.9 **(51.2–58.5)**	1213	41.8 **(38.2–45.6)**	449	15.5 **(12.8–18.6)**	72	2.5 **(1.7–3.5)**	692	23.9 **(20.5–27.6)**	2899
35–49 years	1266	46.7 **(43.0–50.4)**	1397	51.5 **(47.9–55.2)**	854	31.5 **(27.6–35.6)**	71	2.6 **(1.9–3.7)**	472	17.4 **(14.8–20.4)**	2711
Level of education											
Never attended school	2465	55.0 **(51.0–59.0)**	1908	42.6 **(38.7–46.6)**	1024	22.9 **(19.6–26.5)**	98	2.2 **(1.6–3.0)**	785	17.5 **(14.6–20.9)**	4481
Primary	520	55.4 **(51.3–59.5)**	384	40.9 **(36.6–45.4)**	120	12.8 **(10.0–16.3)**	21	2.3 **(1.3–4.0)**	243	25.8 **(22.2–29.9)**	939
Secondary	972	58.6 **(55.5–61.6)**	614	37.0 **(34.0–40.1)**	173	10.4 **(8.5–12.7)**	44	2.7 **(1.8–3.9)**	397	23.9 **(21.5–26.6)**	1659
Higher education	213	58.8 **(52.2–65.0)**	127	35.1 **(28.8–41.9)**	12	3.4 **(1.8–6.6)**	6	1.7 **(0.7–3.8)**	109	30.0 **(23.6–37.3)**	362
Wealth quintile											
First	845	55.9 **(51.5–60.3)**	634	41.9 **(37.6–46.3)**	282	18.6 **(15.4–22.3)**	31	2.1 **(1.2–3.5)**	321	21.2 **(17.0–26.1)**	1512
Second	1035	56.6 **(52.5–60.6)**	750	41.0 **(37.0–45.2)**	291	15.9 **(13.2–19.1)**	52	2.9 **(1.8–4.5)**	407	22.3 **(18.9–26.1)**	1828
Third	1055	59.3 **(55.0–63.6)**	668	37.6 **(33.4–41.9)**	317	17.9 **(14.6–21.6)**	31	1.7 **(1.0–3.1)**	320	18.0 **(14.6–22.0)**	1778
Fourth	851	55.7 **(51.1–60.3)**	612	40.1 **(35.5–44.8)**	271	17.7 **(14.7–21.2)**	37	2.4 **(1.5–3.8)**	304	19.9 **(16.1–24.4)**	1527
Fifth	385	48.3 **(43.7–52.9)**	369	46.2 **(42.0–50.6)**	169	21.2 **(16.8–26.3)**	18	2.3 **(1.3–3.9)**	182	22.8 **(19.1–27.0)**	798
Caste/ethnic group											
Brahmin/Chhetri	993	54.2 **(50.4–58.0)**	772	42.1 **(38.0–46.4)**	355	19.3 **(15.5–23.9)**	42	2.3 **(1.5–3.6)**	375	20.5 **(17.0–24.4)**	1833
Terai Madheshi other caste	665	60.4 **(53.8–66.7)**	405	36.7 **(31.0–42.9)**	296	26.9 **(21.0–33.8)**	6	0.5 **(0.2–1.2)**	102	9.3 **(5.4–15.5)**	1101
Dalit	565	59.1 **(54.4–63.6)**	359	37.6 **(33.1–42.3)**	160	16.7 **(12.7–21.7)**	21	2.2 **(1.3–3.9)**	178	18.6 **(14.8–23.1)**	956
Newar	75	40.0 **(31.4–49.2)**	109	58.0 **(48.6–66.9)**	33	17.7 **(10.9–27.3)**	2	1.1 **(0.2–4.8)**	74	39.2 **(30.4–48.8)**	188
Janajati	1552	53.0 **(49.3–56.6)**	1282	43.8 **(40.1–47.5)**	445	15.2 **(12.0–19.0)**	94	3.2 **(2.2–4.6)**	743	25.4 **(22.4–28.6)**	2929
Muslim	263	87.9 **(79.3–93.3)**	29	9.7 **(5.0–18.1)**	11	3.7 **(1.6–8.1)**	1	0.4 **(0.1–1.9)**	17	5.6 **(2.4–12.6)**	299
Other	57	42.3 **(32.9–52.2)**	77	56.7 **(47.4–65.6)**	30	22.0 **(15.7–30.1)**	2	1.5 **(0.3–7.6)**	45	33.2 **(24.1–43.8)**	136
Time taken to nearest Government health facility											
<30 minutes	1425	57.2 **(52.2–62.0)**	983	39.4 **(34.8–44.2)**	477	19.1 **(16.1–22.6)**	61	2.5 **(1.5–3.9)**	445	17.8 **(14.5–21.7)**	2493
30–60 minutes	1124	53.1 **(49.2–57.0)**	919	43.4 **(39.4–47.6)**	462	21.8 **(18.1–26.1)**	55	2.6 **(1.6–4.2)**	403	19.0 **(15.8–22.7)**	2117
>60 minutes	1623	57.3 **(53.4–61.0)**	1131	39.9 **(36.0–44.0)**	391	13.8 **(10.9–17.4)**	53	1.9 **(1.3–2.8)**	686	24.2 **(21.0–27.8)**	2834
Ecological zone											
Mountain	253	47.1 **(39.7–54.7)**	255	47.4 **(39.3–55.6)**	106	19.6 **(10.1–34.6)**	14	2.5 **(1.3–5.0)**	136	25.2 **(19.0–32.6)**	538
Hill	1940	58.3 **(54.1–62.3)**	1324	39.8 **(35.4–44.3)**	388	11.7 **(8.6–15.6)**	91	2.7 **(1.7–4.3)**	845	25.4 **(21.8–29.3)**	3328
Terai	1979	55.3 **(50.8–59.8)**	1455	40.7 **(36.6–44.9)**	836	23.4 **(20.2–26.9)**	65	1.8 **(1.2–2.6)**	553	15.5 **(12.7–18.7)**	3577

Note: Numbers may not sum to total due to rounding.

**Table 2:** Inequalities in the use of modern family planning methods: findings from binomial and multinomial logistic regression.

	Any modern method		Permanent methods		Long-acting reversible methods		Short-acting methods	
	Crude OR (95% CI)	Adjusted OR (95% CI)	Crude OR (95% CI)	Adjusted OR (95% CI)	Crude OR (95% CI)	Adjusted OR (95% CI)	Crude OR (95% CI)	Adjusted OR (95% CI)
Age-group								
15–24 years	1	1	1	1	1	1	1	1
24–34 years	2.3 (1.9–2.9)	2.5 (2.0–3.1)∗	12.3 (7.1–21.3)	10.8 (6.3–18.6)∗	1.7 (1.0–2.7)	1.8 (1.1–3.0)∗	1.2 (0.9–1.5)	1.4 (1.1–1.8)∗
35–49 years	3.5 (2.8–4.4)	3.7 (2.9–4.8)∗	30.9 (17.8–53.7)	25.6 (14.7–44.7)∗	1.8 (1.0–3.0)	2.0 (1.2–3.5)∗	0.8 (0.6–1.0)	1.0 (0.7–1.4)
Level of education								
Never attended school	1	1	1	1	1	1	1	1
Primary	0.9 (0.7–1.1)	1.1 (0.9–1.4)	0.4 (0.3–0.6)	0.8 (0.6–0.9)∗	1.0 (0.6–1.8)	1.1 (0.6–1.8)	1.6 (1.2–2.1)	1.4 (1.1–1.8)∗
Secondary	0.7 (0.6–0.9)	1.0 (0.8–1.2)	0.3 (0.3–0.5)	0.6 (0.5–0.8)∗	1.2 (0.8–1.7)	1.3 (0.9–2.0)	1.4 (1.1–1.8)	1.3 (0.9–1.6)
Higher education	0.7 (0.5–0.9)	1.1 (0.7–1.5)	0.1 (0.0–0.2)	0.2 (0.1–0.5)∗	0.7 (0.3–1.8)	0.9 (0.4–2.2)	2.0 (1.4–2.7)	1.8 (1.1–2.6)∗
Wealth quintile								
First	1	1	1	1	1	1	1	
Second	0.9 (0.8–1.1)	0.9 (0.8–1.1)	0.8 (0.6–1.0)	0.9 (0.7–1.2)	1.3 (0.7–2.5)	1.2 (0.7–2.1)	1.0 (0.8–1.3)	0.9 (0.7–1.2)
Third	0.8 (0.6–1.0)	0.8 (0.7–1.0)	0.9 (0.7–1.2)	1.1 (0.8–1.5)	0.8 (0.4–1.6)	0.7 (0.4–1.4)	0.8 (0.5–1.1)	0.7 (0.5–0.9)∗
Fourth	0.9 (0.7–1.1)	0.9 (0.7–1.1)	0.9 (0.7–1.2)	0.9 (0.7–1.2)	1.1 (0.5–2.3)	1.0 (0.5–2.1)	0.9 (0.6–1.3)	0.9 (0.6–1.3)
Fifth	1.1 (0.9–1.5)	1.0 (0.8–1.3)	1.1 (0.8–1.6)	1.0 (0.7–1.6)	1.1 (0.5–2.1)	1.1 (0.5–2.2)	1.0 (0.7–1.5)	1.0 (0.7–1.6)
Caste/ethnic group								
Brahmin/Chhetri	1	1	1	1	1	1	1	1
Terai Madhesi other castes	0.7 (0.5–1.0)	0.7 (0.5–0.9)∗	1.5 (1.0–2.3)	0.8 (0.5–1.3)	0.2 (0.0–0.5)	0.2 (0.1–0.7)∗	0.3 (0.2–0.7)	0.6 (0.3–1.0)
Dalit	0.8 (0.6–1.0)	0.9 (0.7–1.1)	0.8 (0.5–1.2)	0.7 (0.4–1.1)	0.9 (0.4–1.9)	1.0 (0.6–2.2)	0.8 (0.6–1.2)	1.1 (0.8–1.5)
Newar	1.8 (1.2–2.7)	1.9 (1.4–2.7)∗	0.8 (0.4–1.6)	0.9 (0.5–1.6)	0.4 (0.0–2.3)	0.4 (0.1–2.2)	2.5 (1.7–3.6)	2.6 (1.8–3.7)∗
Janajati	1.0 (0.8–1.3)	1.2 (0.9–1.4)	0.7 (0.5–1.0)	0.7 (0.5–0.9)∗	1.4 (0.8–2.3)	1.6 (0.9–2.7)	1.3 (1.0–1.7)	1.5 (1.1–1.9)∗
Muslim	0.1 (0.0–0.3)	0.2 (0.1–0.3)∗	0.1 (0.0–0.3)	0.1 (0.1–0.2)∗	0.1 (0.0–0.8)	0.2 (0.1–1.1)	0.2 (0.0–0.5)	0.3 (0.1–0.8)∗
Other	1.7 (1.1–2.7)	2.1 (1.4–3.2)∗	1.1 (0.7–1.9)	1.5 (0.8–2.9)	0.6 (0.1–3.4)	0.5 (0.1–3.8)	1.9 (1.1–3.1)	1.9 (1.2–3.4)∗
Time taken to nearest government health facility								
<30 minutes	1		1	1	1	1	1	1
30–60 minutes	1.1 (0.9–1.4)	1.1 (0.9–1.4)	1.1 (0.8–1.5)	1.3 (0.9–1.8)	1.0 (0.5–2.0)	0.9 (0.5–1.8)	1.0 (0.8–1.3)	0.9 (0.8–1.2)
>60 minutes	1.0 (0.7–1.3)	1.0 (0.8–1.3)	0.6 (0.4–0.9)	1.0 (0.7–1.4)	0.7 (0.4–1.3)	0.5 (0.3–0.9)∗	1.4 (1.0–1.9)	1.1 (0.8–1.4)
Ecological zone								
Mountain	1		1	1	1	1	1	1
Hill	0.7 (0.5–1.0)	0.7 (0.5–0.9)∗	0.5 (0.2–1.2)	0.4 (0.2–1.3)	1.0 (0.4–2.5)	1.3 (0.5–3.1)	1.0 (0.6–1.5)	0.9 (0.6–1.4)
Terai	0.7 (0.5–1.1)	1.0 (0.7–1.3)	1.2 (0.5–2.7)	1.4 (0.5–3.6)	0.7 (0.3–1.5)	0.9 (0.4–2.1)	0.5 (0.3–0.8)	0.7 (0.5–1.1)

*P < 0.05.
